# Supplementary material for: Electrical detection and modulation of magnetism in a Dy-based ferroelectric single-molecule magnet
Source: Nat Commun. 2023 Nov 30;14:7901. doi: 10.1038/s41467-023-43815-w (PMC10689763; doi:10.1038/s41467-023-43815-w)
Supplement: Supplementary file 1 — Supplementary Information [file 41467_2023_43815_MOESM1_ESM.pdf]

Supplementary Information

Electrical detection and modulation of magnetism in a Dy-based ferroelectric single-molecule magnet

Yu-Xia Wang[1#], Dan Su[2#], Yinina Ma[2], Young Sun[2,3]*, Peng Cheng[1]*

[1]Key Laboratory of Advanced Energy Material Chemistry, Frontiers Science Center for New Organic Matter, Renewable Energy Conversion and Storage Center, and Haihe Laboratory of Sustainable Chemical Transformations (Tianjin), College of Chemistry, Nankai University, Tianjin 300071, China

[2]Beijing National Laboratory for Condensed Matter Physics, Institute of Physics, Chinese Academy of Sciences, Beijing 100190, China

[3]Department of Applied Physics and Center of Quantum Materials and Devices, Chongqing University, Chongqing 401331, China

*Corresponding authors. Email: youngsun@cqu.edu.cn; pcheng@nankai.edu.cn

[#]These authors contributed equally to this work.

Contents

1. **Basic characterization** ··· **pages 4-8**

Crystal photos (Supplementary Fig. 1)

Thermogravimetric analysis (Supplementary Fig. 2)

Single crystal structures (Supplementary Fig. 3)

Variable-temperature luminescence spectra (Supplementary Fig. 4)

Pyroelectric current (Supplementary Fig. 5)

2. **Additional crystal structure descriptions** ··· **page 9**

3. **Magnetic relaxation characterization** ··· **pages 10-12**

Temperature-dependent of the AC magnetic susceptibility (Supplementary Fig. 6)

Frequency-dependent of the AC magnetic susceptibility (Supplementary Fig. 7)

Magnetic relaxation analyses (Supplementary Fig. 8)

4. **Ab initio calculations** ··· **pages 13-15**

Additional crystal structure descriptions

Ab initio calculated electronic states (Supplementary Fig. 9)

Principal magnetic axis of the ground Kramers' doublet (Supplementary Fig. 10)

5. **Additional magnetic relaxation analyses** ··· **pages 16-17**

6. **Magnetic anisotropy** ··· **pages 18-19**

M-T and *M-H* anisotropies of the single-crystal sample (Supplementary Fig. 11)

Magnetic rotation measurements (Supplementary Fig. 12)

7. **Magnetodielectric effect** ··· **pages 20-21**

Dielectric permittivity and loss tangent as a function of magnetic field (Supplementary Fig. 13)

Magnetodielectric effect with after applied *E*-field poling (Supplementary Fig. 14)

8. **Electric-field control of magnetization** ··· **pages 22-31**

Magnetization measurement probe (Supplementary Fig. 15)

Magnetoelectric anisotropy (Supplementary Fig. 16)

Magnetic and electric phase zones (Supplementary Fig. 17)

E-field control of *M*−*H* loop at different temperatures (Supplementary Fig. 18)

E-field control of temperature-dependent AC magnetic susceptibility (Supplementary Fig. 19)

E-field control of frequency-dependent AC magnetic susceptibility (Supplementary Fig. 20)

Magnetic relaxation analyses under *E*-field (Supplementary Fig. 21)

E-field control of magnetization relaxation time at 2 K (Supplementary Fig. 22)

Magnetization relaxation time at different temperatures without *E*-field (Supplementary Fig. 23)

9. Excluding heating effect with applied electric fields··············pages 32-34

Discussions on excluding heating effect

Sample temperature fluctuations as the *E*-field off and on (Supplementary Fig. 24)

E-field control of *M*−*H* loops with ITO films (Supplementary Fig. 25)

10. Tables···pages 35-41

Crystallographic data and structural refinements (Supplementary Table 1)

Continuous shape measure calculated values (Supplementary Table 2)

Magnetic relaxation fitting results for a powder sample (Supplementary Table 3)

Magnetic relaxation fitting results for a single-crystal sample (Supplementary Tables 4-5)

SA-CASSCF/RASSI- calculated electronic states (Supplementary Table 6)

Magnetic relaxation fitting results for a single-crystal sample under *E*-fields (Supplementary Table 7)

1. Basic characterization

a

b

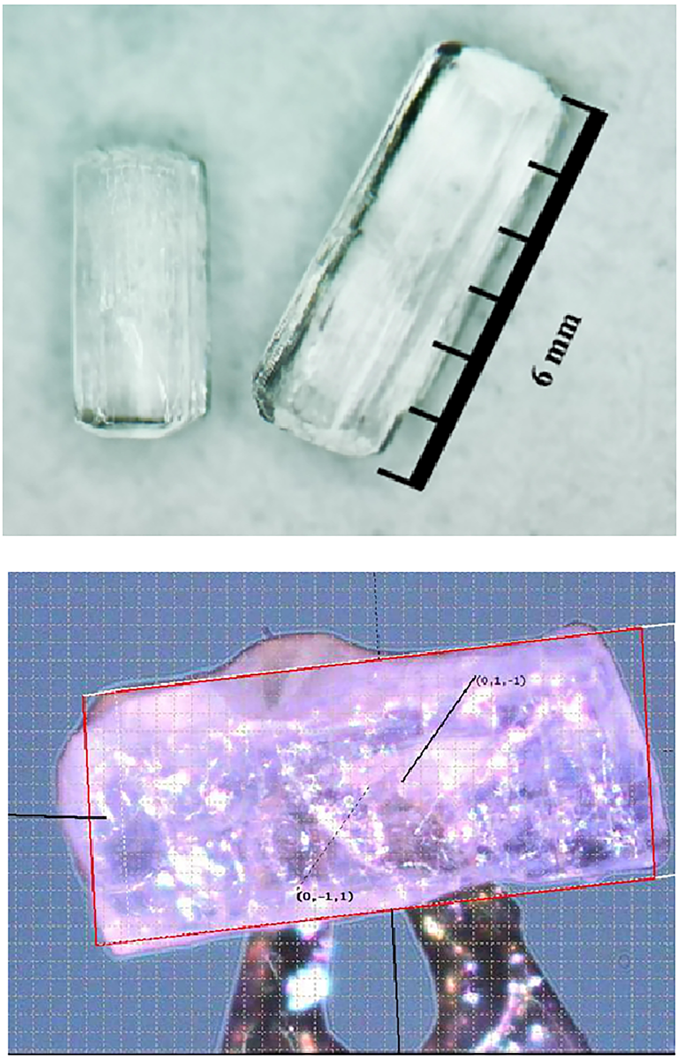

Supplementary Fig. 1 | Photographs of single crystals. **a**, the representative crystals with size scales. **b**, the (0-11) plane, as determined by single-crystal X-ray diffraction.

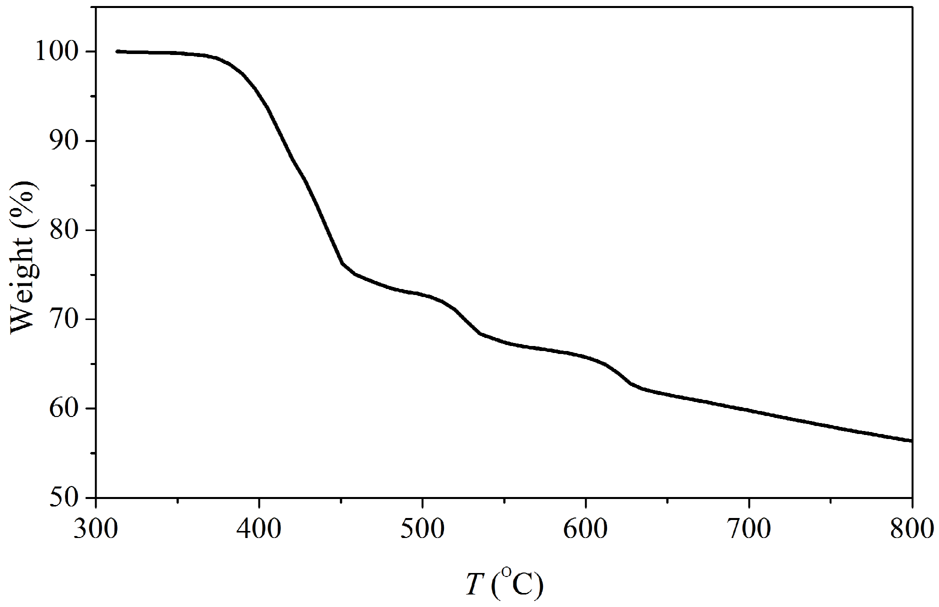

Supplementary Fig. 2 | Thermogravimetric analysis of Dy-SMM.

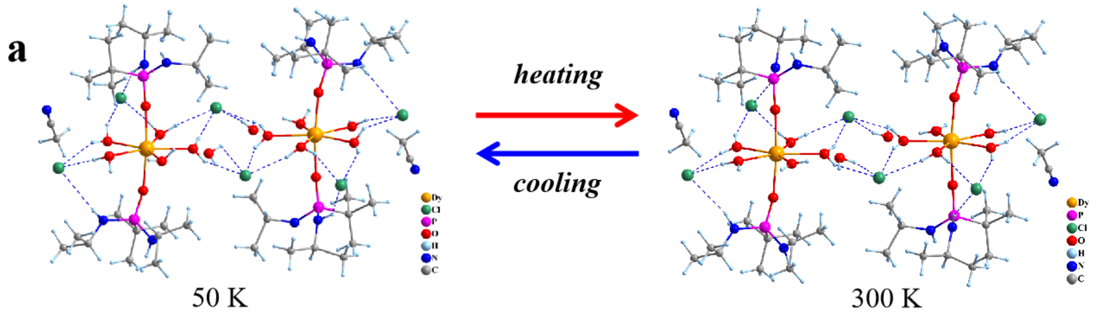

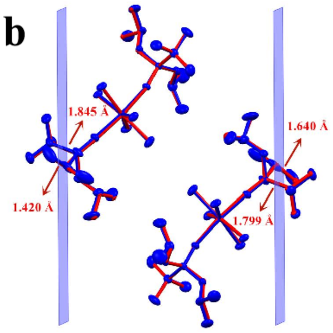

Bonds	50K			300K
P-C	1.845(16) Å (P4-C31)	1.799(17) Å (P1-C1)	1.822(17) Å (average)	1.823(6) Å (P2-C11)
C-C	1.420(2) Å (C35-C37)	1.640(2) Å (C5-C7)	1.530(2) Å (average)	1.533(11) Å (C15-C16)

Supplementary Fig. 3 | Crystal structures of Dy-SMM. a, Hydrogen bonds in the structures at 50 and 300 K. **b**, Overlay image of the molecular units at 50 K (blue) and 300 K (red). The labelled bond lengths are the P–C and C–C bonds at 50 K. The faint blue planes represent the symmetry element of the *c* glide plane in the structure at 300 K. The specific bond lengths at both temperatures are listed on the right.

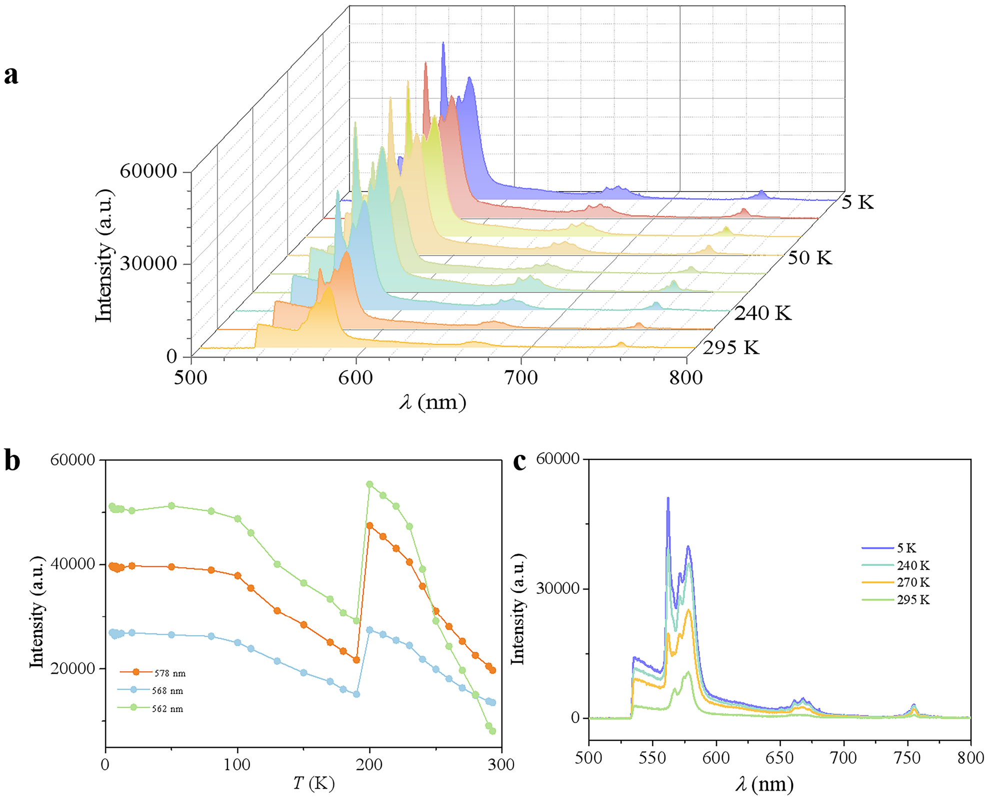

Supplementary Fig. 4 | Variable-temperature luminescence spectra of Dy-SMM. **a**, Luminescence spectra in the temperature range of 5-295 K. **b**, Luminescence intensity at λ = 578 nm, 568 nm and 562 nm illustrating anomalies near the ferroelectric-to-paraelectric phase transition. **c**, Comparison of the luminescence spectra at 5 K, 240 K, 260 K and 295 K. The main peak shifted from 568 nm to 578 nm, indicating that the dominant energy transfer transition changed.

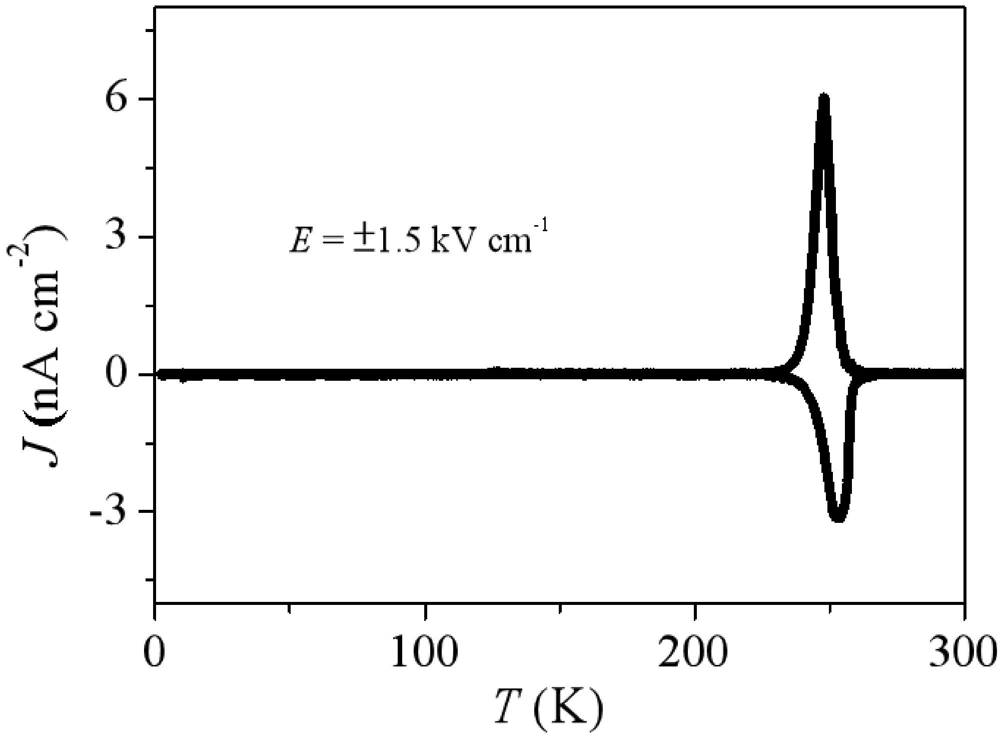

Supplementary Fig. 5 | Pyroelectric currents of Dy-SMM as a function of temperature. The peaks at approximately 250 K indicate a ferroelectric phase transition. The pyroelectric current was collected with increasing temperature at a warming rate of 2 K/min. The amplifying part of the low temperatures show no other peaks. The electric fields were applied perpendicular to the (0-11) plane.

2. Additional crystal structure descriptions

Variable-temperature crystal structures illustrated a structural transition. At 300 K, one crystalline-independent Dy^{3+} ion was observed in each symmetric unit, with two coordinated ligands in opposite directions, five coordinated water molecules in the equatorial plane, three Cl^{-} ions, one water molecule and one acetonitrile molecule in the outer coordination sphere (Supplementary Fig. 3).

The average bond lengths of the three nearby diagonal substituent groups at 50 K were found to be equal to the corresponding bond lengths at 300 K, causing the *c* glide planes to vanish. The differences between the structures at 300 and 50 K are similar to those between the two conformational isomers, and the slight lengthening or shortening of the bonds rather than significant displacement is mainly due to the steric hindrance of the ligand.

The coordination environment of the Dy^{3+} ion was found to be a slightly distorted pentagonal bipyramid with average Dy–O distances of 2.206(1) Å (axial) and 2.376(4) Å (equatorial) and an axial O–Dy–O angle of 173.10(13) (Supplementary Table 1). Continuous shape measure (CSM) calculations were carried out on the DyO_7 sites. The minimum deviation from an ideal model of the D_{5h} geometry indicated that the Dy^{3+} ion was situated within a slightly distorted pentagonal bipyramid coordination environment at both 300 K and 50 K (Supplementary Table 2). Therefore, **Dy-SMM** is expected to have high-performance SMM properties due to exhibiting the appropriate uniaxial anisotropy.

3. Magnetic relaxation characterization

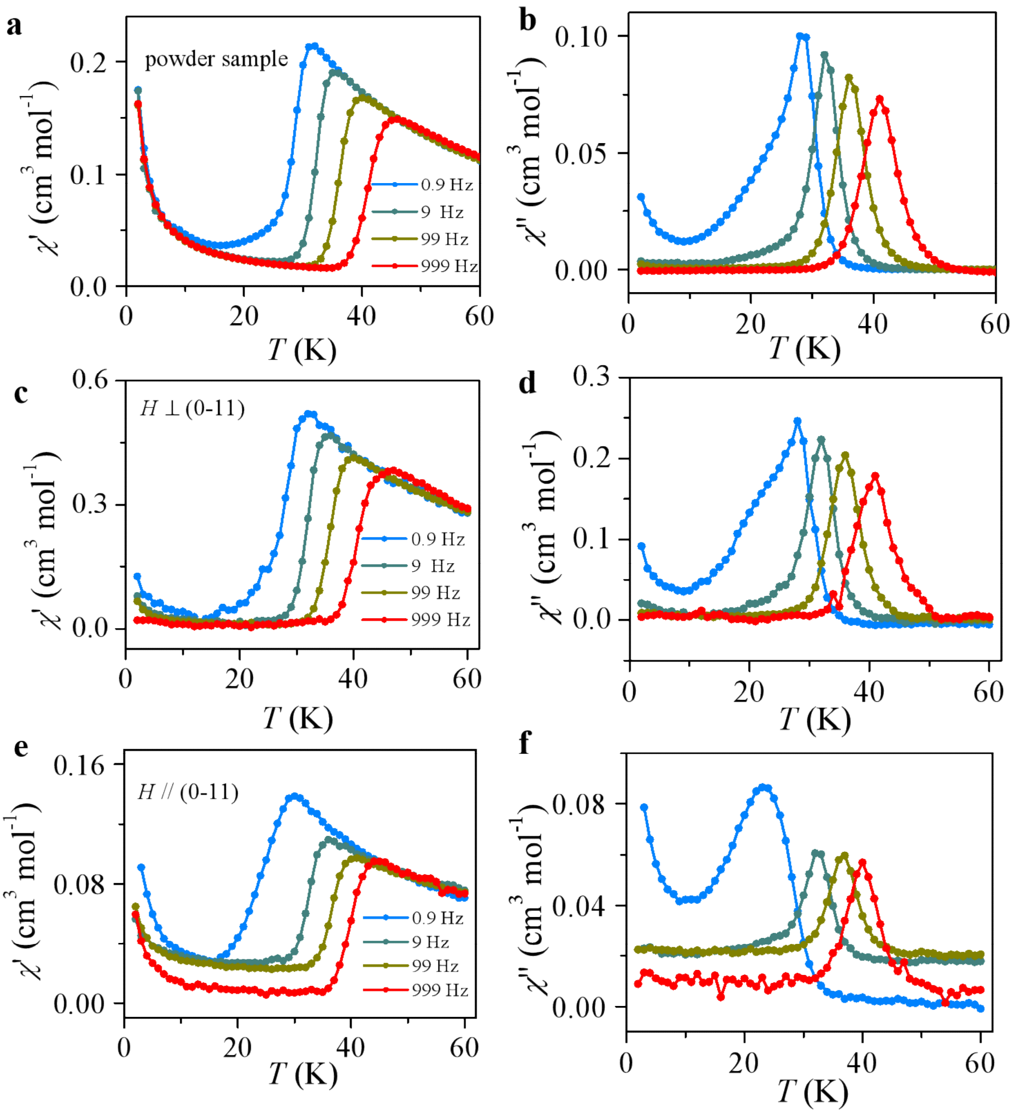

Supplementary Fig. 6 | Temperature dependence of the AC magnetic susceptibility of Dy-SMM. a,b Temperature dependence of the in-phase (χ') and out-of-phase (χ'') components of the AC magnetic susceptibility of a powder sample. **c-f** Temperature dependence of the in-phase (χ') and out-of-phase (χ'') components of the AC magnetic susceptibility of a single-crystal sample with a magnetic field applied (**c**,**d**) perpendicular and (**e**,**f**) parallel to the (0-11) plane.

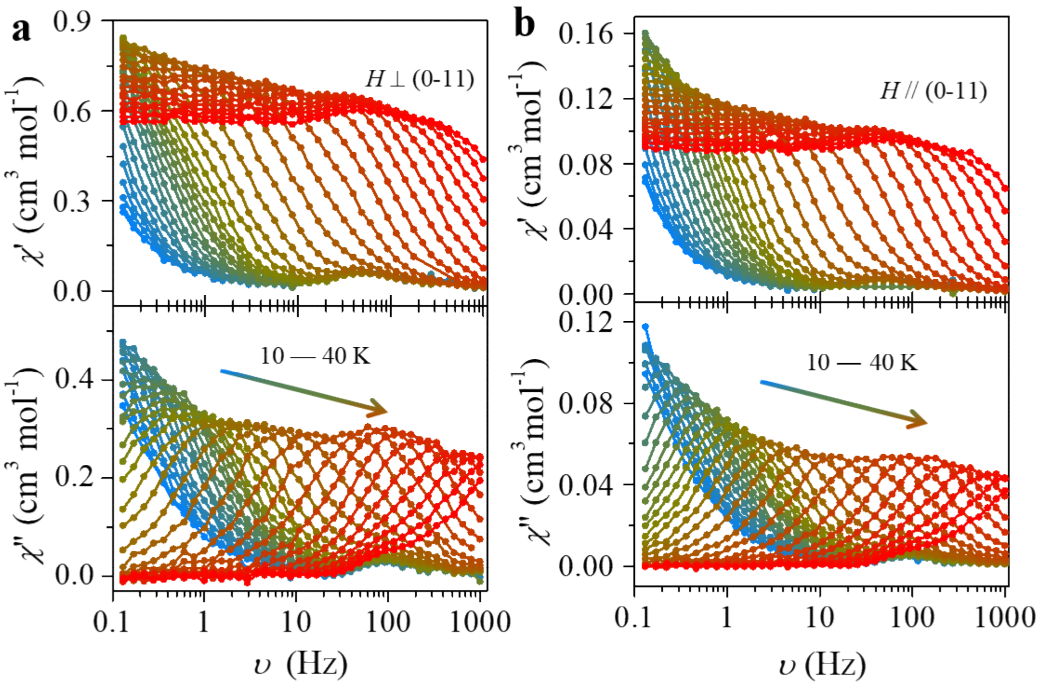

Supplementary Fig. 7 | Frequency dependence of the AC magnetic susceptibility of Dy-SMM. In-phase (χ') and out-of-phase (χ'') components of the AC magnetic susceptibility with the magnetic field applied perpendicular (**a**) and parallel (**b**) to the (0-11) plane. Lines are included as visual guides.

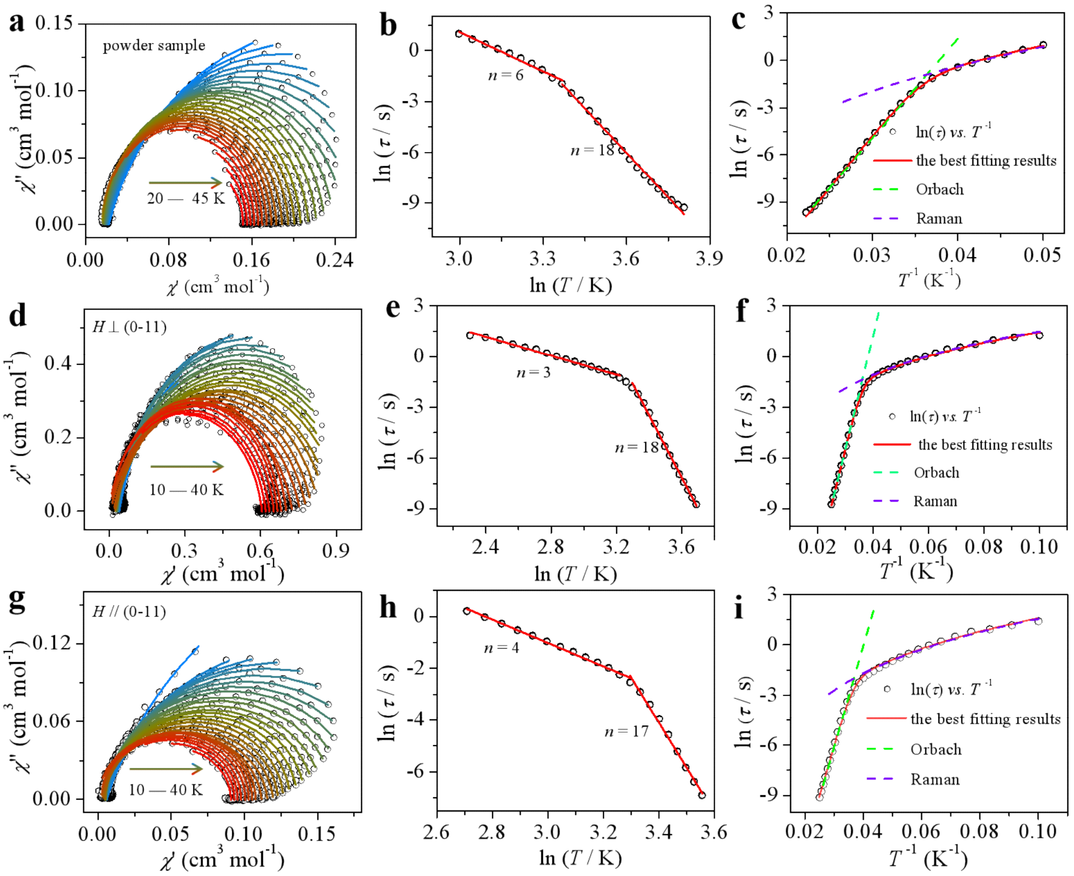

Supplementary Fig. 8 | Magnetic relaxation analyses. a-c Fitted Arrhenius equation for a powder sample. **d-f** Fitted Arrhenius equation for a single-crystal sample with the magnetic field applied perpendicular to the (0-11) plane. **g-i** Fitted Arrhenius equation for a single-crystal sample with the magnetic field applied parallel to the (0-11) plane. **a**, **d**, **g** Cole–Cole plots of the AC magnetic susceptibility. The solid lines represent the best fits to the generalized Debye model. **b**, **e**, **h** The solid red lines represent the best fit to piecewise power law equations. **c**, **f**, **i** The solid red lines represent the best fit to Arrhenius equations, while the dashed lines represent the contributions of the Orbach (green) and Raman (violet) relaxation processes.

4. *Ab initio* calculations

Ab initio calculations were performed at the SA-CASSCF/RASSI level to predict the electronic structure of **Dy-SMM**. The structures obtained with the XRD analysis were employed directly without any optimization in the theoretical calculations. The results showed that the ground state doublet was a pure |±15/2> state (99.3%). Furthermore, the g_z value close to 20 was observed to be almost collinear with the two shortest chemical bonds of the Dy ion (*g* is the Landau factor). The energy of the first excited state was 504 K higher than that of the ground state (98.5% |±13/2> state). The deviation angle of g_z in this state relative to the g_z of the ground Kramers doublet was only 3.4°, still quite close to the pseudo-C_5 axis. The second excited state was at 677 K above the ground state with a highly mixed wavefunction of 39.7%|±1/2> + 35.9%| ∓ 1/2> + 15.1%|±3/2>. The *g* tensor contained a significant transverse component (g_x = 0.52, g_y = 1.91, g_z = 17.87), and the g_z axis was almost perpendicular to the g_z of the ground Kramers doublet (89.6°). Therefore, the magnetic relaxation through the Orbach process likely proceeded via the second excited state, giving a calculated energy barrier of 677 K, which agrees well with the experimentally determined value of 643 K.

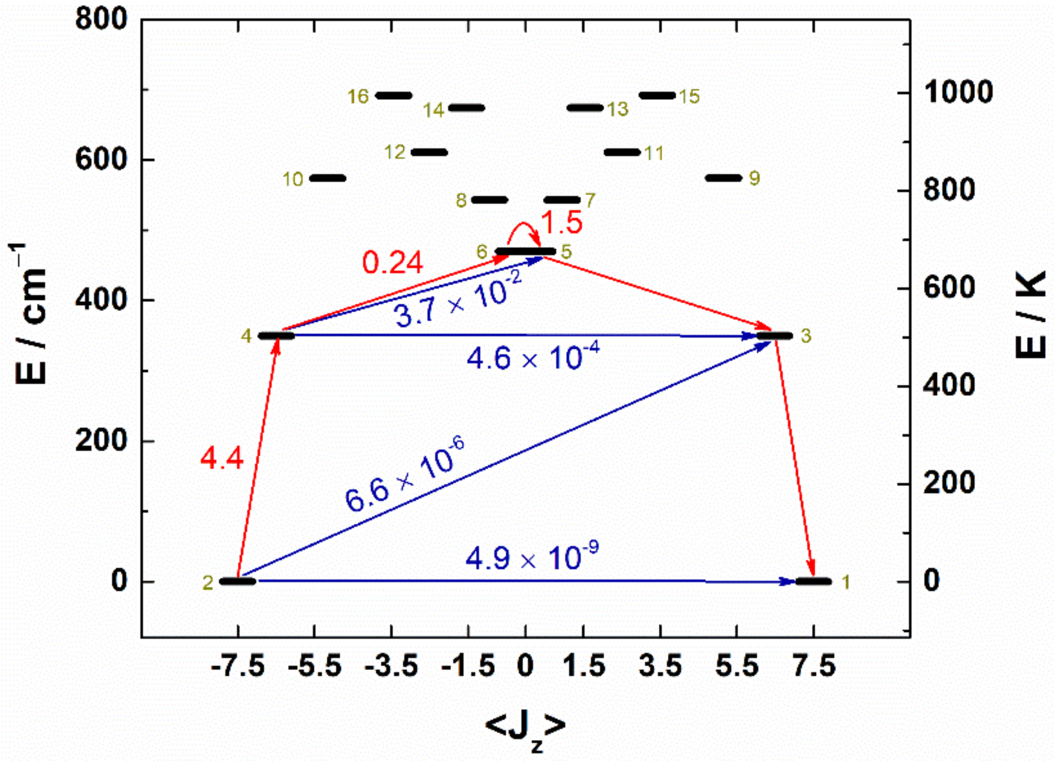

Supplementary Fig. 9 | *Ab initio*-calculated electronic states of $[Dy(L)_2(H_2O)_5]Cl_3{\cdot}H_2O{\cdot}CH_3CN$ with the probability of transition between different substates.

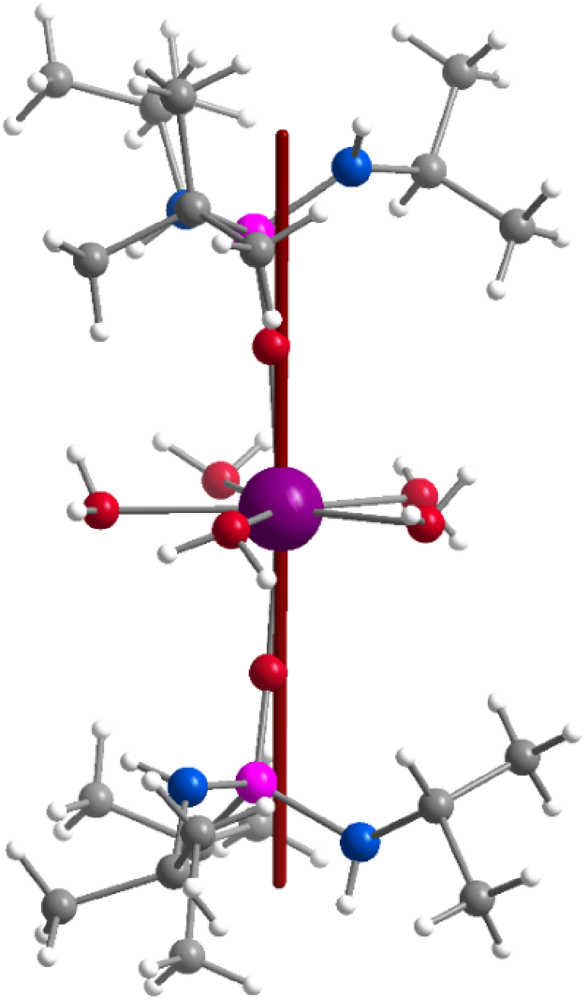

Supplementary Fig. 10 | Molecular structure of $[Dy(L)_2(H_2O)_5]Cl_3{\cdot}H_2O{\cdot}CH_3CN$ as calculated by *ab initio* calculations. The red line is the principal magnetic axis of the ground Kramers doublet.

5. Additional descriptions of magnetic relaxation analyses

Magnetic relaxation processes of the single-crystal sample of **Dy-SMM** were investigated through fitting the Cole-Cole plot by the generalized Debye model and fitting the temperature dependent relaxation time plot by the equation including of the Orbach and Raman relaxation processes.

The small values of the coefficient α (< 0.17) under the application of a magnetic field (H) both perpendicular and parallel to the (0-11) plane (Supplementary Fig. 8 and Supplementary Tables 4 and 5) indicated a narrow distribution for the relaxation time (τ). The best fitting results showed that ln(τ) was inversely proportional to T, indicating that the thermally activated Orbach process was dominant in the higher temperature range (29−40 K). In the lower temperature range (10−28 K), however, the temperature-dependent relaxation tended to obey the power law $\tau \sim T^{-n}$ instead, presumably due to the involvement of the Raman process below 29 K (as is commonly observed in SMMs with extremely long relaxation times).

By including the equations of the Orbach and Raman relaxation processes, the temperature dependent relaxation time plots were described well by $\tau^{-1} = \tau_0^{-1}\exp(-U_{eff}/k_BT) + CT^n$ (τ_0 is the preexponential factor, U_{eff} is the effective energy barrier, C and n are constant parameter values of the Raman process that do not have a physical basis, and k_B is the Boltzmann constant) over the whole temperature range. This yielded U_{eff} = 643(4) and 601(1) K, τ_0 = 1.6(7)×10^{-11} and 3.3 (9)×10^{-11} s, C = 4.5(7)×10^{-4} and 5.7(3)×10^{-5} s^{-1}K^{-n}, and n = 2.7(2) and 3.5(6) for H perpendicular and parallel to the (0-11) plane, respectively. In addition, the *ab initio* calculations yielded

an energy barrier of 677 K (Supplementary Table 6), which agrees well with the experimentally determined values and illustrates that the Orbach process likely proceeded via the second excited state (Supplementary Fig. 9). Hence, the above results of ferroelectric and SMM properties indicated that **Dy-SMM** is a ferroelectric SMM.

6. Magnetic anisotropy

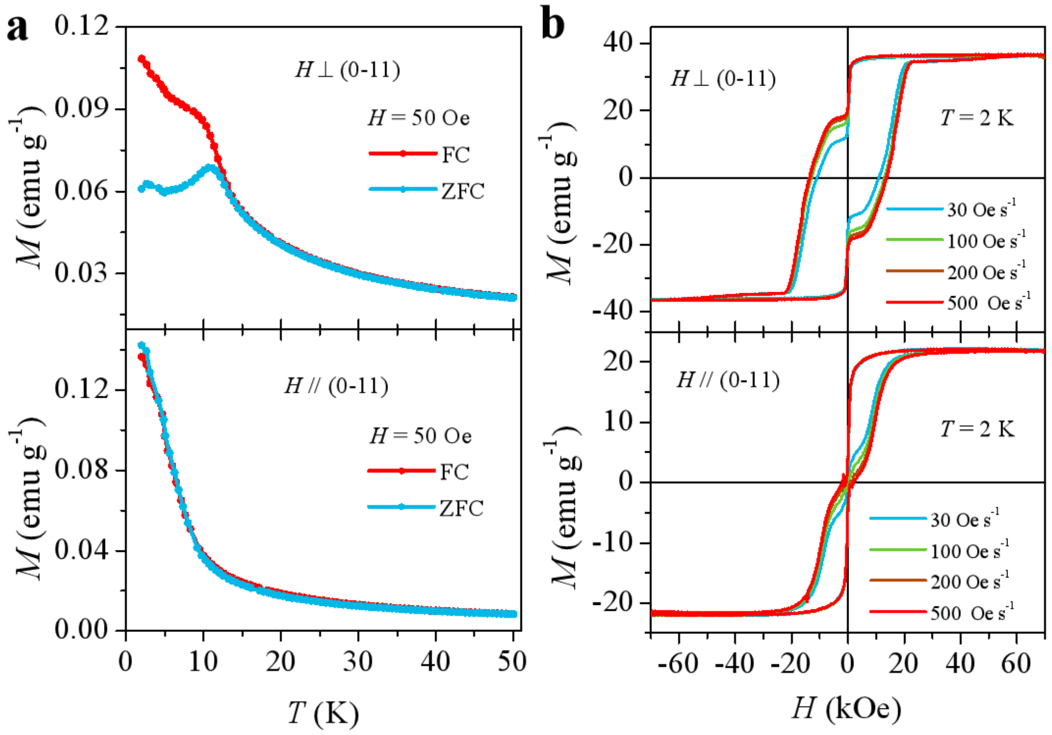

Supplementary Fig. 11 | Magnetic anisotropy of a single-crystal sample of Dy-SMM with magnetic fields applied perpendicular and parallel to the (0-11) plane. a, ZFC and FC magnetization as a function of temperature for a single-crystal sample. **b**, Magnetic hysteresis loops at 2 K with different sweep rates.

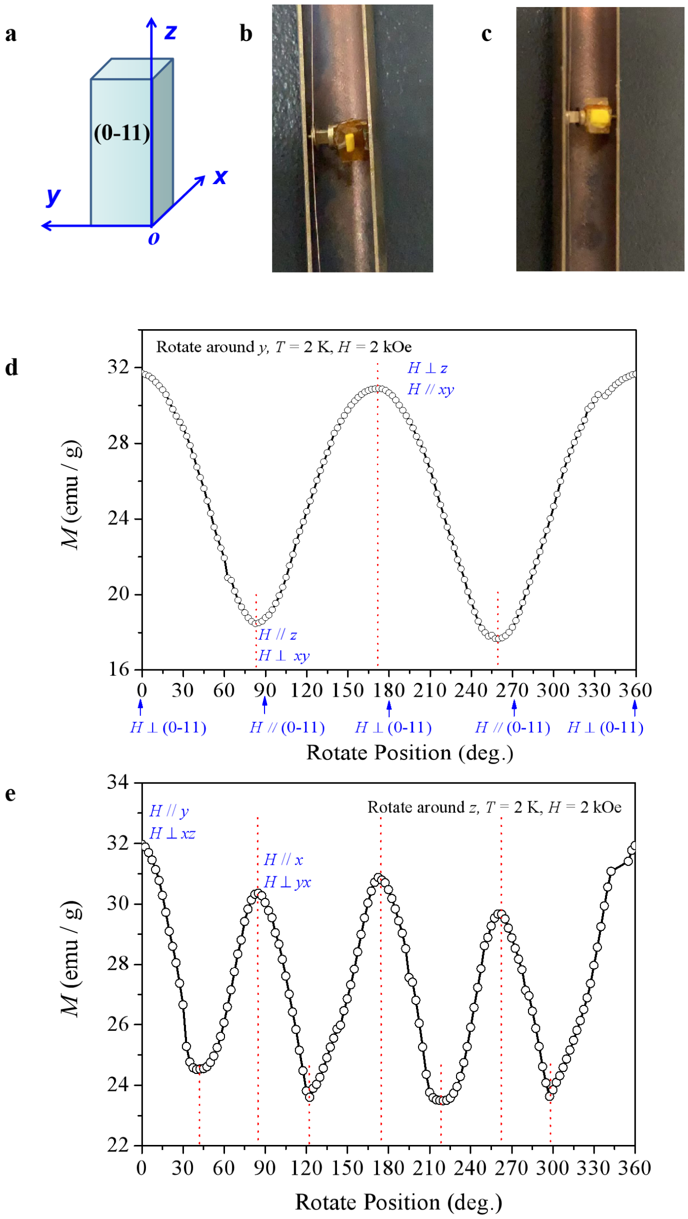

Supplementary Fig. 12 | Magnetization as a function of crystal rotation at 2 K under a magnetic field of 2 kOe. a, Morphology of the single crystal of **Dy-SMM**. **b**, and **c**, Pictures of the sample position in the rotation experiments. **d**,**e** Angular dependence of magnetization with the rotation around *z* and *y* axis, respectively.

7. Magnetodielectric effect

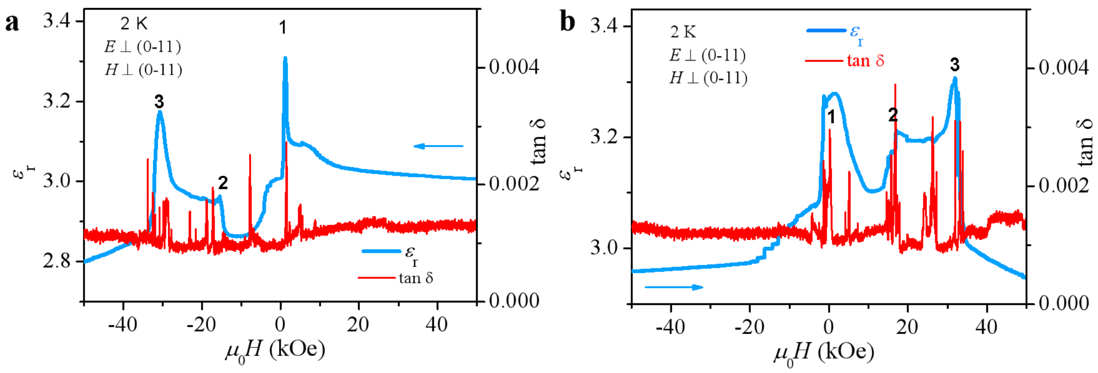

Supplementary Fig. 13 | Magnetodielectric effect. Dielectric permittivity and loss tangent with both the electric and magnetic fields perpendicular to the (0-11) plane at f = 20 kHz as a function of decreasing (**a**) and increasing (**b**) magnetic field at 2 K. The arrows indicate the direction of the sweeping magnetic field. Both the dielectric permittivity and the loss tangent exhibit clear peaks at the positions of resonant quantum tunnelling of magnetization.

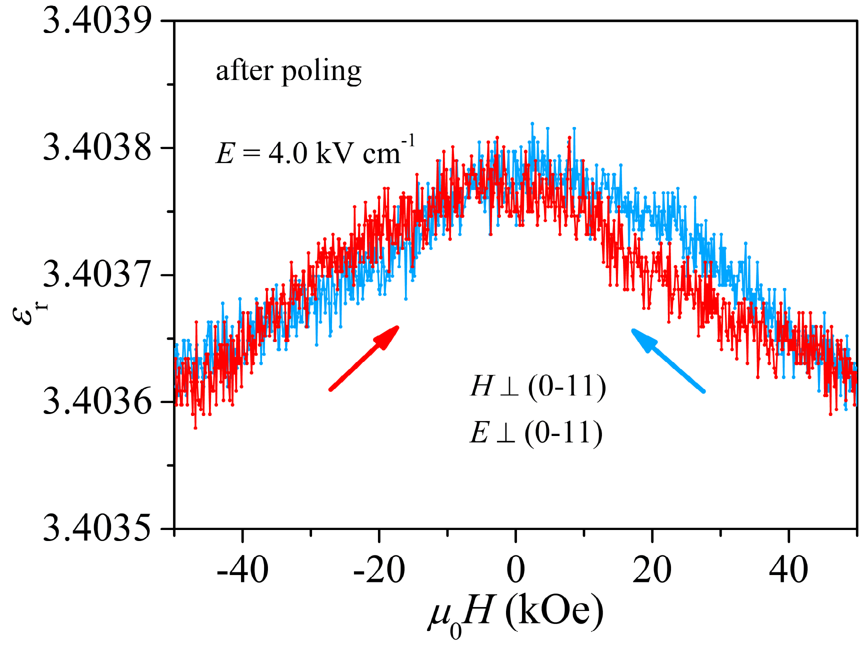

Supplementary Fig. 14 | Magnetodielectric effect after an *E*-field poling. Dielectric permittivity as a function of magnetic field after poling with an electric field E = 4.0 kV/cm. The arrows indicate the direction of sweeping of the magnetic field. The blue and red lines indicate magnetic field decreasing and increasing, respectively. After poling with an *E*-field of 4 kV/cm from 300 to 2 K, the MD behaviour detected as 2 K was completely changed.

8. Electric-field control of magnetization

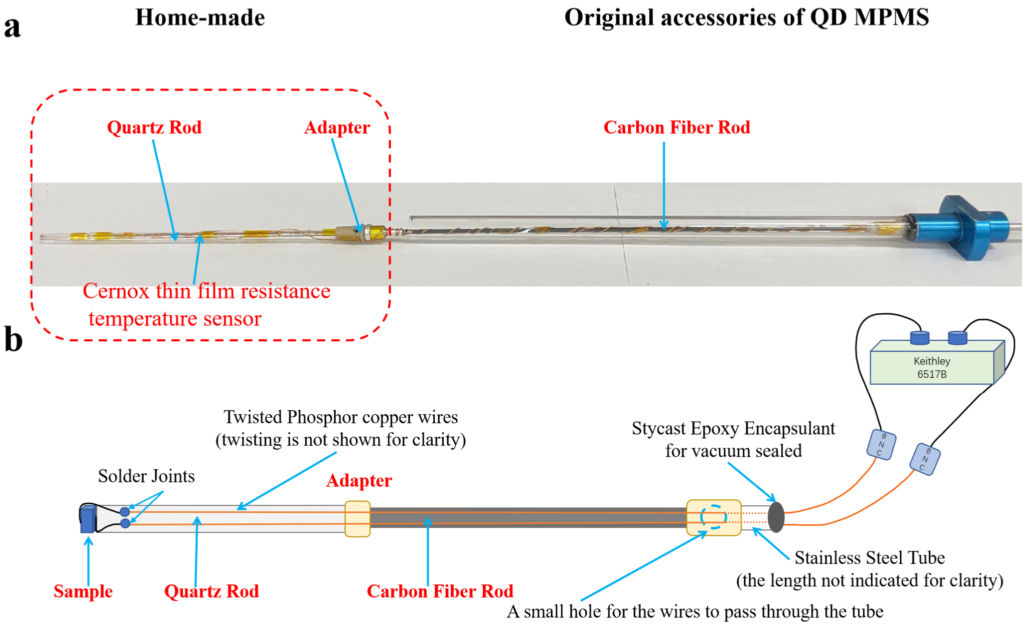

Supplementary Fig. 15 | A probe for magnetization measurements under electric fields. **a**, the picture of the probe. **b**, A schematic of the probe with twisted phosphor copper wires.

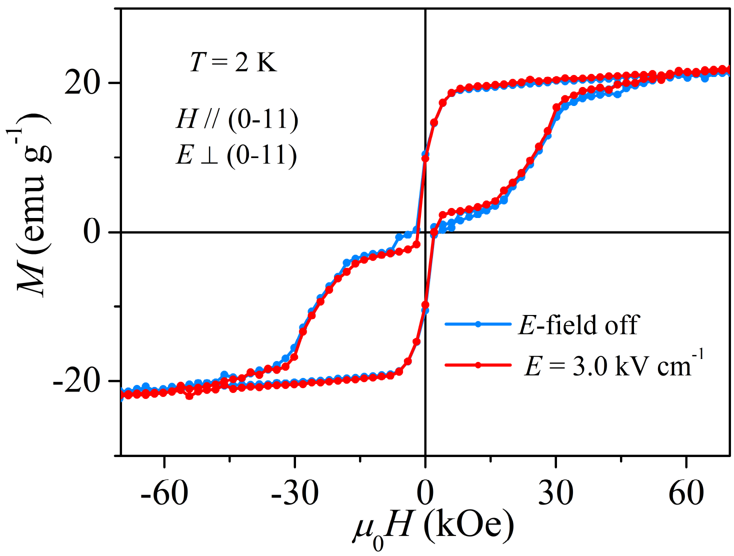

Supplementary Fig. 16 | Anisotropy of the electric field control of magnetization. The *M*–*H* loops were collected at 2 K with a magnetic field applied parallel to the (0-11) plane and an *E*-field applied perpendicular to the (0-11) plane. The sample was cooled from 300 to 2 K. When the temperature was stable at 2 K, the *M*-*H* loop with *E*-field off (E = 0 kV cm^{-1}) was firstly measured. Then, turning on the *E*-field, and the *M*-*H* loop was measured under a *E*-field of 3 kV cm^{-1}. In this magnetization direction, the *E*-field had little influence on the *M*-*H* loop. The tremendous anisotropic behaviour strongly suggests that the ME effect is intrinsic.

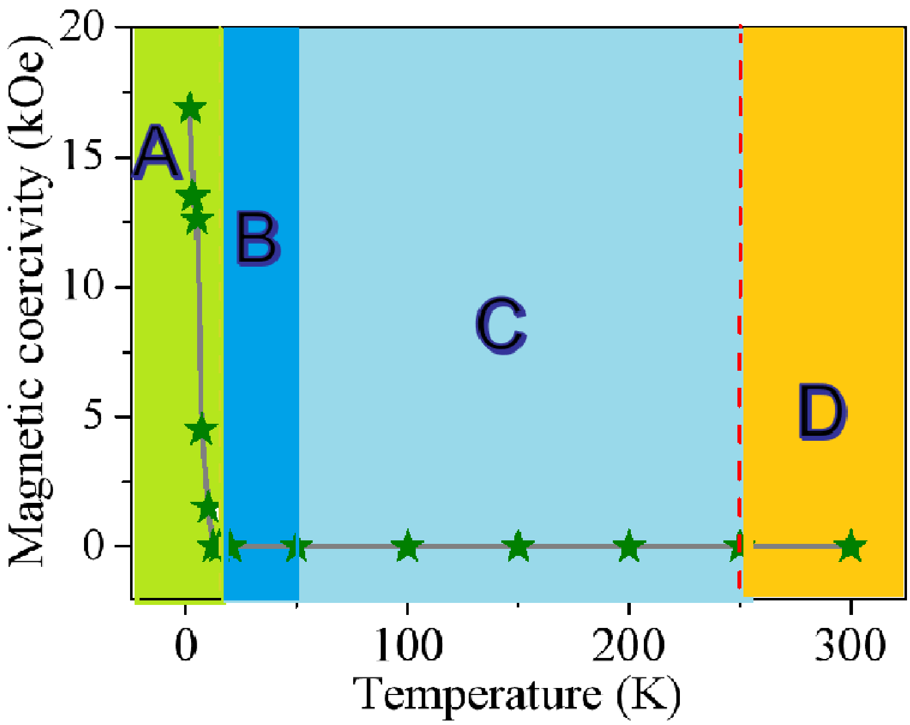

Supplementary Fig. 17 | Magnetic and electric phases in different temperature zones between 2 K and 300 K. H_C is measured at different temperatures without *E*-fields. Region A correlates with the ferroelectric phase accompanied by apparent magnetic hysteresis loops below 12 K. Region B correlates with the ferroelectric phase accompanied by magnetization relaxation behaviours but without hysteresis loops between 12 K and 50 K. Region C correlates with the ferroelectric phase accompanied by paramagnetic behaviours between 50 K and 250 K. Region D correlates with the paraelectric phase accompanied by paramagnetic behaviours.

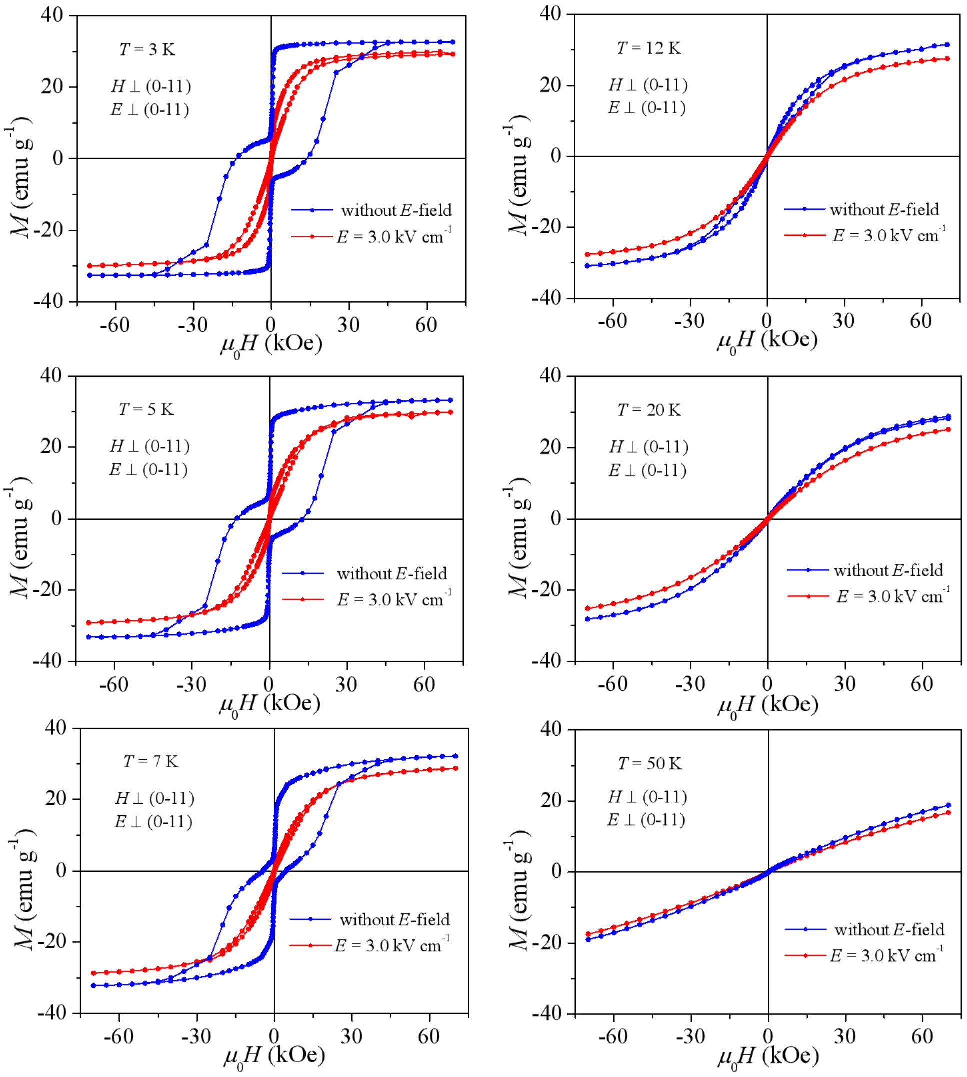
T = 3 K
H ⊥ (0-11)
E ⊥ (0-11)
without E-field
E = 3.0 kV cm-1
T = 12 K
T = 5 K
T = 20 K
T = 7 K
T = 50 K
M (emu g-1)
μ0H (kOe)

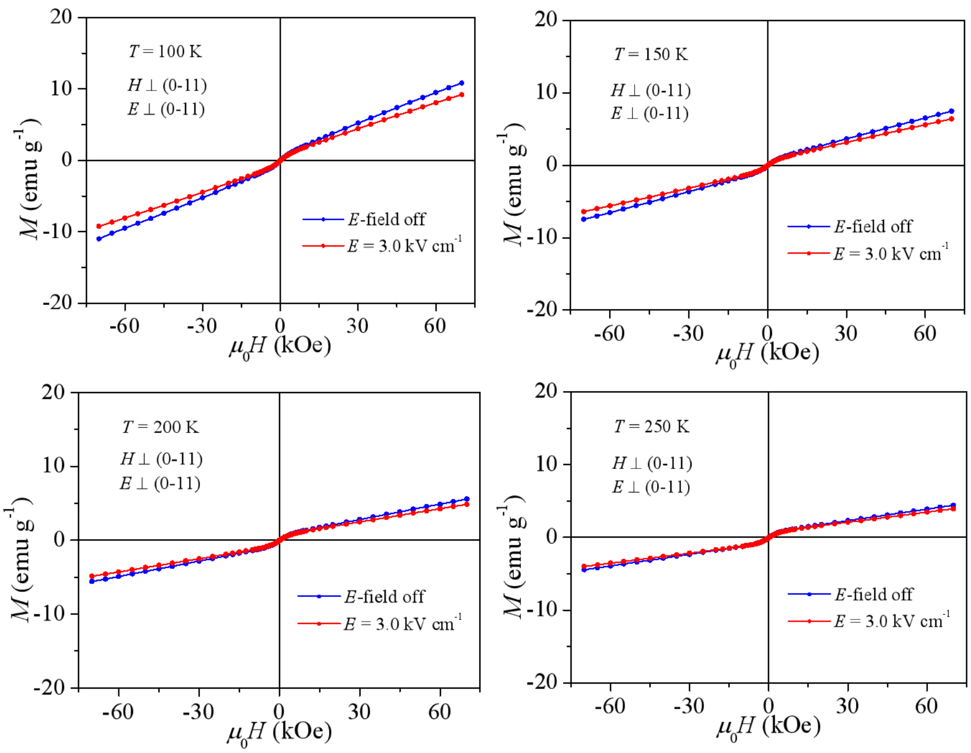

Supplementary Fig. 18 | Temperature dependence of electric-field control of magnetization. Comparisons of the *M*−*H* loops with and without an *E*-field at different temperatures. First, the *M*−*H* loops with *E*-field off were collected at fixed temperatures between 2 K and 300 K. Then, turning on the *E*-field, the *M*−*H* loops were measured with an *E*-field of 3 kV/cm between 2 K and 300 K.

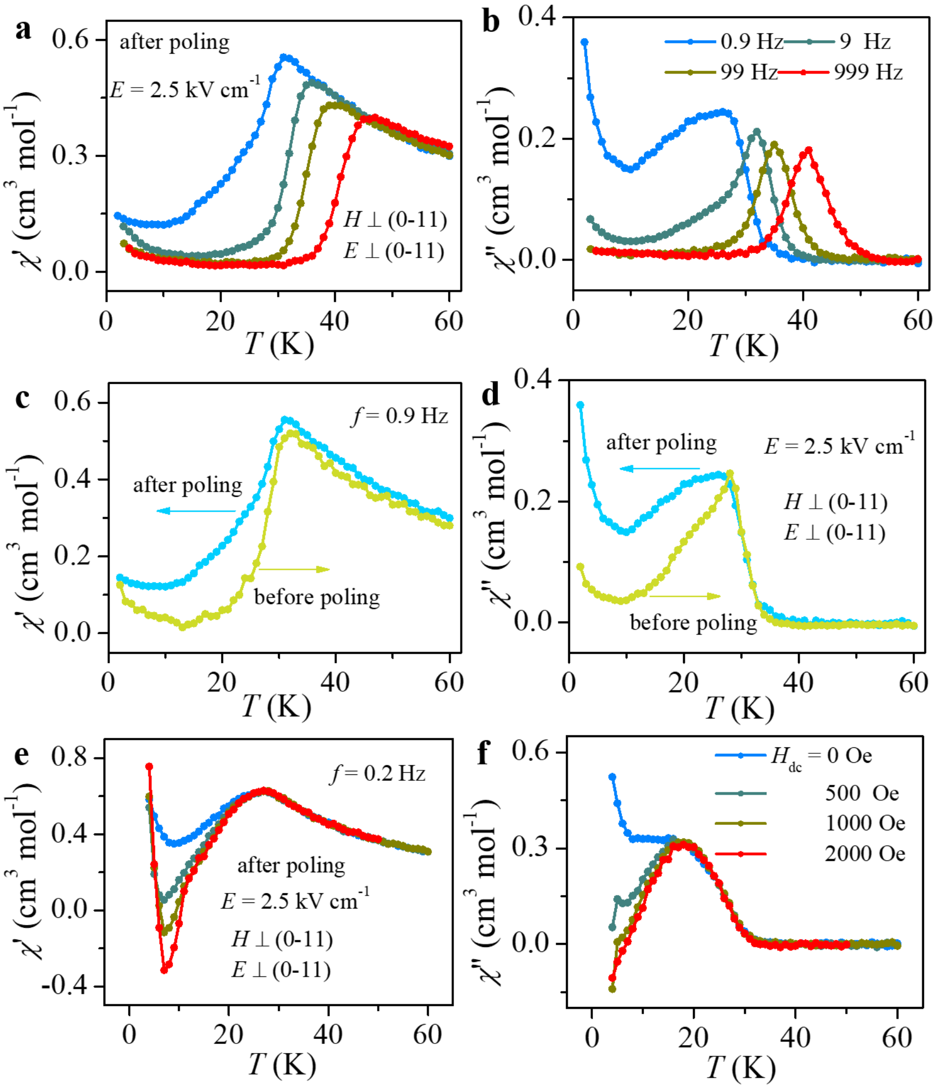

Supplementary Fig. 19 | AC magnetic susceptibility for a single-crystal sample of Dy-SMM with *E*-field. The magnetic and electric fields were applied perpendicular to the (0-11) plane. a,**b** Temperature dependence of the in-phase (χ') and out-of-phase (χ'') components of the AC magnetic susceptibility. **c**,**d** Comparison of the temperature dependence of the in-phase (χ') and out-of-phase (χ'') components of the AC susceptibility at 0.9 Hz. The rapid increase in the AC magnetic susceptibility at low temperatures after *E*-field poling was ascribed to the enhancement of the RQTM. **e**,**f** Temperature dependence of the AC magnetic susceptibility at 0.2 Hz after poling under different DC bias magnetic fields. The increase in χ'' at low temperatures rapidly decreases with increasing DC magnetic field, corresponding to suppression of the RQTM under DC bias magnetic fields.

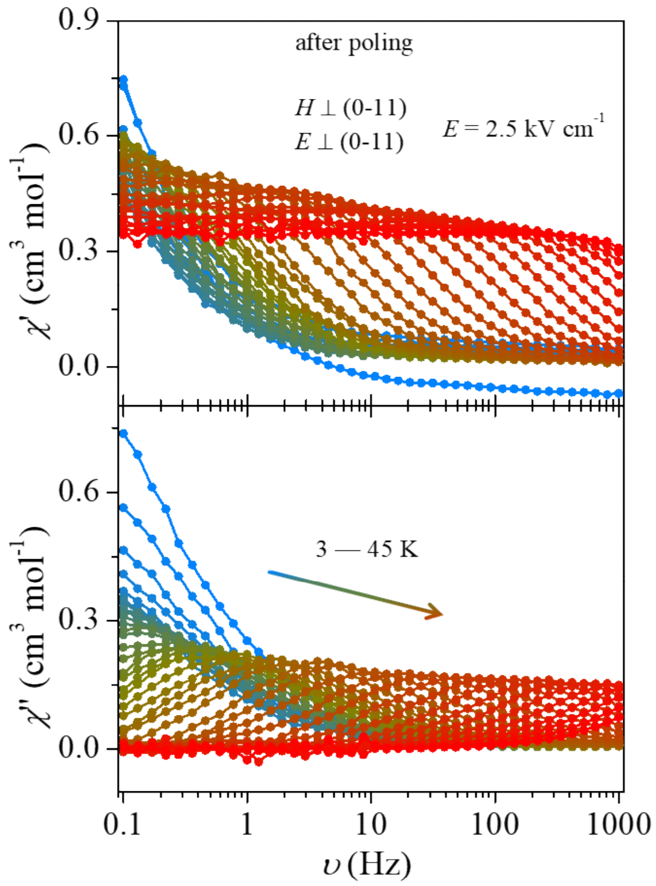

Supplementary Fig. 20 | Frequency dependence of the in-phase (χ') and out-of-phase (χ'') components of the AC magnetic susceptibility after *E*-field poling. The magnetic and electric fields were applied perpendicular to the (0-11) plane. The peaks in the high temperature range under an electric field are similar to those without *E*-field poling. The results suggest that the *E* field has little influence on the thermal relaxation processes.

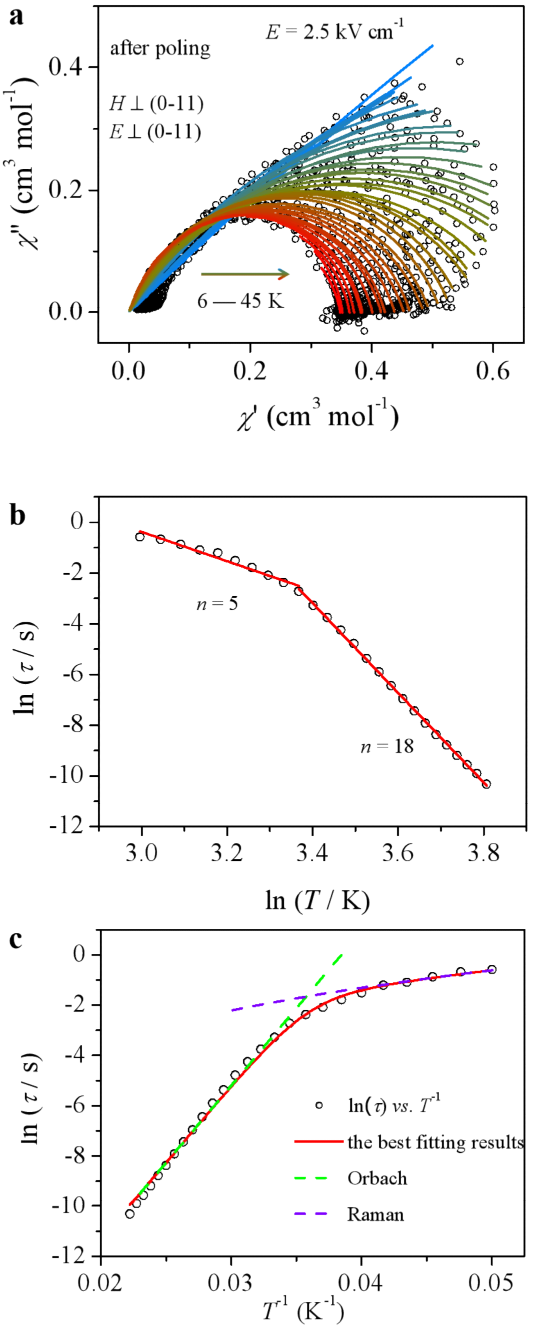

Supplementary Fig. 21 | Magnetic relaxation analyses for a single-crystal sample of Dy-SMM after *E*-field poling. a Best fit results for a powder sample. **b**,**c** Best fit results. The magnetic and electric fields were applied perpendicular to the (0-11) plane. The results further identify that the thermal relaxation processes are slightly disturbed by external electric fields.

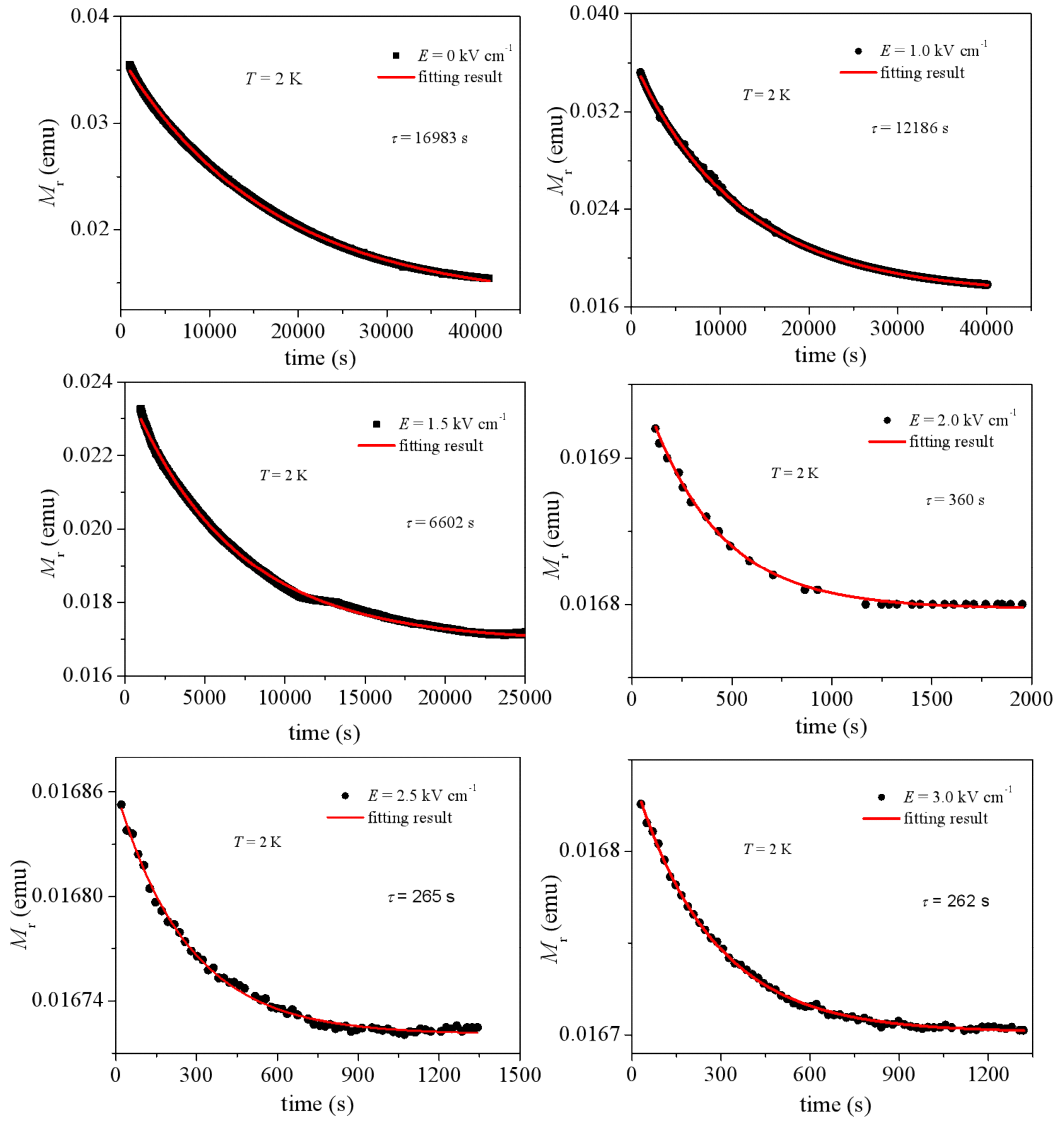

Supplementary Fig. 22 | Magnetization relaxation under different electric fields at 2 K for a single-crystal sample Dy-SMM. The solid lines represent the best fits. The magnetic and electric fields were applied perpendicular to the (0-11) plane. Both the relaxation time and the remanent magnetization clearly decrease with increasing electric field.

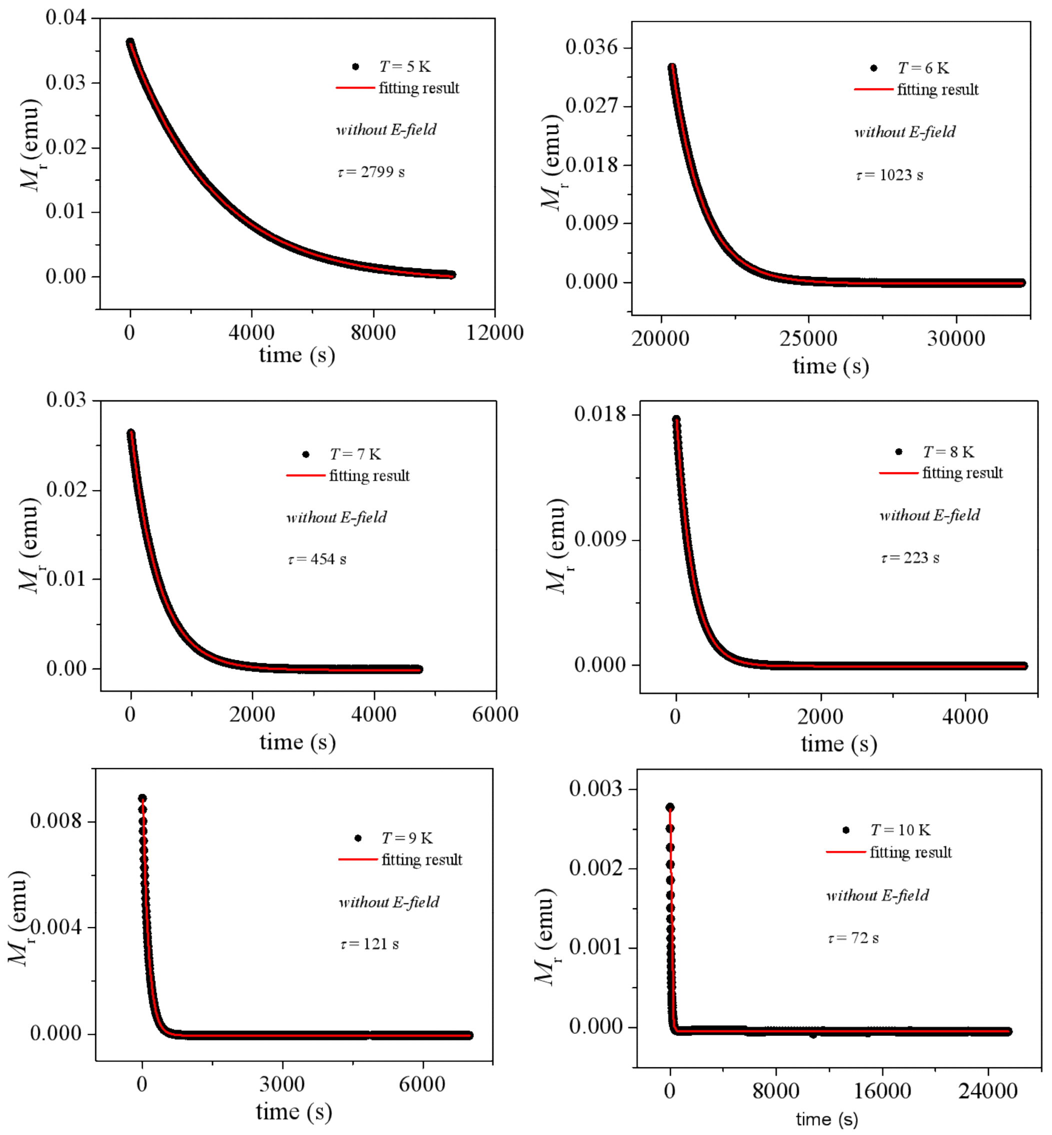

Supplementary Fig. 23 | Magnetization relaxation for a single-crystal sample Dy-SMM without electrodes at 5, 6, 7, 8, 9, and 10 K. The solid lines represent the best fits. The magnetic and electric fields were applied perpendicular to the (0-11) plane. Both the relaxation time and the remanent magnetization clearly decrease with increasing temperature.

9. Excluding heating effect with applied electric fields

One may argue that the induced magnetism change could be due to a thermal heating effect when an E-field is applied to the sample. We have safely excluded the heating effect due to the following reasons. Firstly, the multiferroic SMM is highly insulating, with a resistance of ~ 10^{14} ohm and a loss tangent of ~ 0.002 at low temperatures. The applied E-fields of several kV/cm only generate a tiny current of 10^{-11} ampere at 2 K. Secondly, we monitored the sample temperature through a Cernox thin film resistance temperature sensor placed near the sample during the magnetization measurements, and observed a maximum temperature fluctuation of approximately 0.006 K (Supplementary Fig. 24). This is far below the temperature change that would be necessary to produce heat-induced changes in magnetization. Thirdly, we performed a control experiment by placing the sample between two parallel conductive indium tin oxide (ITO) films without electrical contact with the sample. There is no current passing through the sample so that the heating effect can be completely avoided. The ME effect obtained in this non-contacting method is similar to that using the contacting electrodes (Supplementary Fig. 25). There are also other issues to exclude the heating effect. For example, both the magnetic coercivity and remanent magnetization were reduced by the applied E-field, but the saturation magnetization is almost unchanged. The strongly anisotropic behaviour of E-field control of magnetization also suggests that the observed ME effect is intrinsic to the multiferroic SMM.

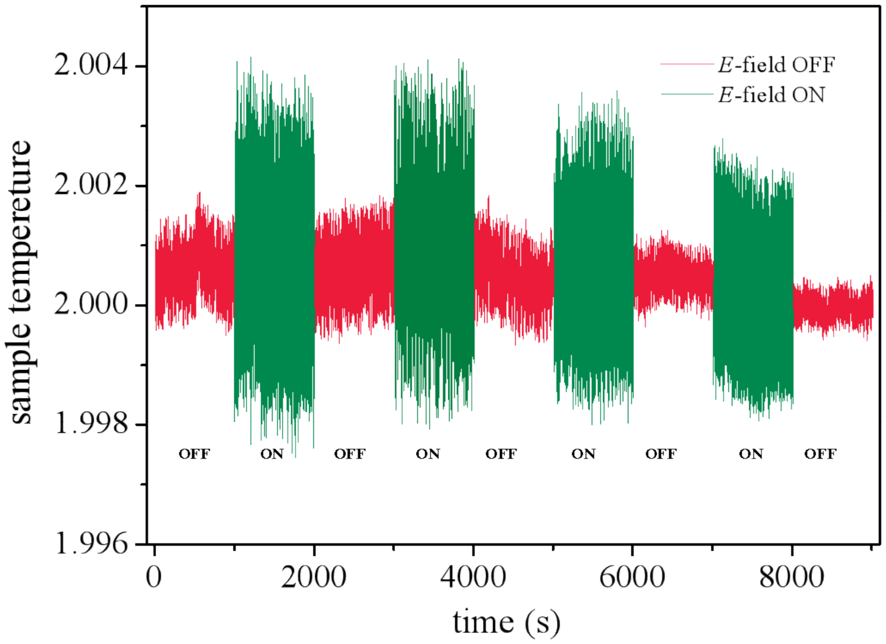

Supplementary Fig. 24 | Sample temperature fluctuations with the *E*-field off and on. A Cernox thin film resistance temperature sensor (Lake Shore) was placed on the sample to obtain the temperature measurements. The monitored temperature fluctuations are below 0.006, indicating that the sample temperature is maintained in the range of 1.997 K to 2.004 K. Therefore, the change in the *M*-*H* loop under an applied *E*-field cannot originate from the heating effect.

a

b

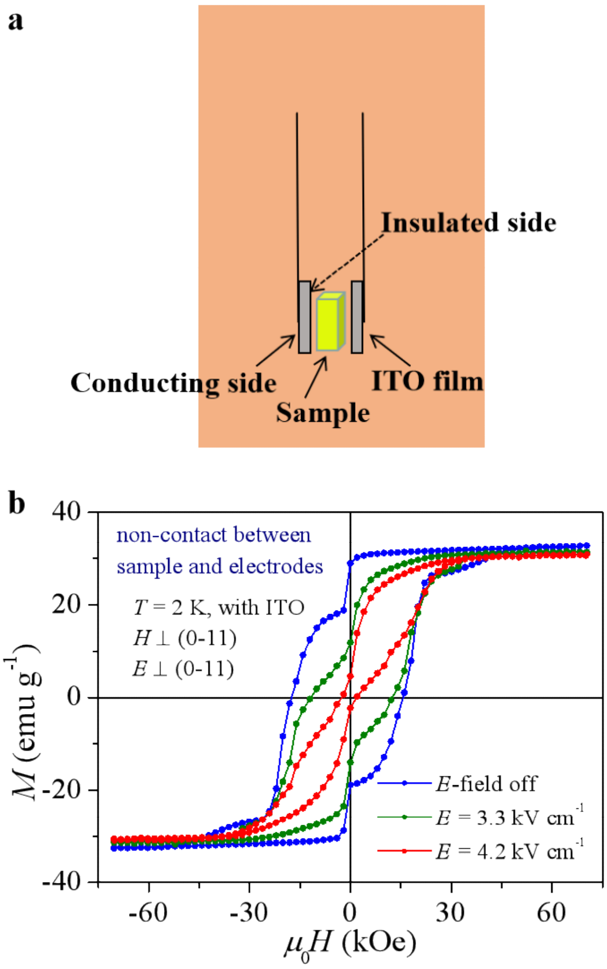

Supplementary Fig. 25 | A control experiment of the ME effect. a, An alternative method of applying electric fields between two indium-tin oxide (ITO) films without direct contact with the sample. This method aims to eliminate any possible heating effect. **b**, The sample was cooled from 300 to 2 K. When the temperature was stable at 2 K, the *M*-*H* loop without an *E*-field (E = 0 kV/cm) was first collected. Then, the *M*-*H* loops with *E* fields of 3.3 kV/cm and 4.2 kV/cm were measured. The ME effect measured in this way is similar to that using the contacting silver paste electrodes.

10. Tables

Supplementary Table 1 | Crystallographic data and structural refinements for **Dy-SMM**.

	50 K	200 K	300 K
Formula	$C_{44}H_{130}Cl_6Dy_2N_{10}O_{16}P_4$	$C_{22}H_{65}Cl_3DyN_5O_8P_2$	$C_{22}H_{65}Cl_3DyN_5O_8P_2$
Formula weight	1717.15	858.58	858.58
Crystal system	monoclinic	monoclinic	monoclinic
Space group	$P2_1$	$P2_1$/c	$P2_1$/c
a (Å)	8.9701(13)	8.9767(15)	9.0509(2)
b (Å)	25.410(4)	25.421(5)	25.8143(7)
c (Å)	18.448(3)	18.451(3)	18.7219(5)
$\alpha = \gamma$ (deg.)	90	90	90
β (deg.)	94.479(5)	94.439(6)	94.673(2)
V (Å^3)	4192.1(11)	4197.8(13)	4359.69(19)
Z	2	4	4
Dc (g·cm^{-3})	1.360	1.359	1.308
R_{int}	0.0735	0.0567	0.0628
GOOF	1.036	1.069	1.030
$^aR_1$	0.0274	0.0195	0.0478
$^bwR_2$	0.0702	0.0481	0.0867
$\Delta\rho_{max}$ (e Å^{-3})	0.76	0.80	0.99
$\Delta\rho_{min}$ (e Å^{-3})	-0.51	-0.50	-0.73
Flack	-0.50(2)	---	---

[a] $R_1 = \sum||Fo| - |Fc||/\sum|Fo|$; [b] $wR_2 = [\sum w(Fo^2 - Fc^2)^2/\sum w(Fo^2)^2]^{1/2}$.

Note: The single-crystal data collected at 200 K in the X-ray diffraction experiment are different from the electrical measurements obtained with the PPMS. The discrepancy could be due to temperature inaccuracy in the single-crystal X-ray diffraction experiments. Because a large cavity is needed to permit CCD operation, the actual temperature of the sample could be well below the controlling temperature, and a long time is required to reach thermal equilibrium.

Supplementary Table 2 | CSM calculations for the DyO_7 sites in **Dy-SMM**.

Structure $[ML_7]$	HP-7	HPY-7	PBPY-7	COC-7	CTPR-7	JPBPY-7	JETPY-7
Dy1 (300 K)	33.193	24.040	0.217	7.942	6.183	2.557	24.044
Dy1A (50 K)	32.552	23.796	0.268	8.025	5.967	2.466	23.651
Dy1B (50 K)	33.447	24.089	0.244	7.708	5.968	2.527	24.101

D_{7h} = heptagon, C_{6v} = hexagonal pyramid, D_{5h} = pentagonal bipyramid, C_{3v} = capped octahedron, C_{2v} = capped trigonal prism, D_{5h} = Johnson pentagonal bipyramid J13, and C_{3v} = Johnson elongated triangular pyramid J7.

Supplementary Table 3 | Best fit results using the generalized Debye model for a powder sample.

T (K)	χ_S	χ_T	τ	α
20	0.02193	0.42201	2.68937	0.20261
21	0.02118	0.38576	1.95427	0.18719
22	0.0206	0.35744	1.45869	0.1739
23	0.02003	0.33052	1.10213	0.15953
24	0.01969	0.31065	0.85342	0.14619
25	0.019	0.29418	0.64542	0.13676
26	0.01868	0.27692	0.47227	0.12036
27	0.0182	0.26103	0.32238	0.10343
28	0.01768	0.25002	0.20378	0.0922
29	0.01735	0.23901	0.11791	0.07303
30	0.01681	0.23006	0.0648	0.06241
31	0.01632	0.22187	0.03499	0.05417
32	0.01586	0.21413	0.01872	0.05059
33	0.01555	0.20667	0.01013	0.04523
34	0.01532	0.20063	0.00559	0.04202
35	0.01506	0.19475	0.00317	0.04122
36	0.01502	0.18873	0.00184	0.03753
37	0.01536	0.18333	0.0011	0.03164
38	0.01629	0.17856	6.84623E-4	0.02412
39	0.01759	0.17402	4.27161E-4	0.01829
40	0.02061	0.16947	2.81951E-4	0.00427
41	0.02305	0.16575	1.90465E-4	1.40314E-16
42	0.02848	0.16202	1.31193E-4	5.56257E-17
43	0.03654	0.15831	9.62759E-5	8.92806E-17
44	0.04711	0.15489	7.4196E-5	1.3188E-16
45	0.06394	0.15116	6.28741E-5	1.75498E-16

Supplementary Table 4 | Best fit results using the generalized Debye model for the magnetic relaxations with the magnetic field perpendicular to the (0-11) plane for a single crystal sample of **Dy-SMM**.

T (K)	χ_S	χ_T	τ	α
10	0.03761	1.24391	3.52558	0.15241
11	0.03550	1.24307	3.17040	0.13433
12	0.03519	1.24563	2.68110	0.11257
13	0.03476	1.15837	2.10622	0.09279
14	0.03457	1.12908	1.73645	0.07859
15	0.03109	1.15542	1.54631	0.08873
16	0.03204	1.09902	1.22956	0.07558
17	0.03096	1.05766	1.00435	0.07646
18	0.02965	1.03801	0.84433	0.08188
19	0.02864	1.03615	0.74234	0.10419
20	0.02745	1.01513	0.62597	0.11553
21	0.02509	1.00471	0.54379	0.12401
22	0.02518	0.99325	0.46454	0.13889
23	0.02389	0.98503	0.40824	0.15375
24	0.02247	0.96076	0.34136	0.16186
25	0.02224	0.94324	0.28351	0.16814
26	0.02109	0.92631	0.22382	0.17332
27	0.02230	0.88869	0.15890	0.17209
28	0.02242	0.86561	0.10864	0.16379
29	0.02067	0.83373	0.06345	0.15651
30	0.01784	0.80499	0.03563	0.15635
31	0.01451	0.78166	0.01892	0.16224
32	0.00220	0.75544	0.00961	0.16583
33	5.25414E-16	0.72441	0.00501	0.14213
34	8.96573E-16	0.70126	0.00271	0.10842
35	3.57296E-15	0.68020	0.00159	0.08375
36	5.92036E-15	0.66527	9.65247E-4	0.07569
37	6.53693E-15	0.65062	6.06499E-4	0.08045
38	1.17043E-14	0.63351	3.78834E-4	0.09024
39	1.86336E-14	0.61865	2.43683E-4	0.09106
40	2.33474E-14	0.60386	1.61923E-4	0.08714

Supplementary Table 5 | Best fitting results using the generalized Debye model for the magnetic relaxations with the magnetic field parallel to the (0-11) plane for a single crystal sample of **Dy-SMM**.

T (K)	χ_S	χ_T	τ	α
10	0.00536	0.58759	4.12416	0.16981
11	0.00566	0.31964	3.24806	0.15050
12	0.00548	0.29225	2.53162	0.12506
13	0.00521	0.29334	2.20714	0.11439
14	0.00519	0.26424	1.64904	0.09306
15	0.00517	0.24117	1.23818	0.06871
16	0.00532	0.22656	0.97638	0.05506
17	0.00503	0.21222	0.76342	0.04784
18	0.00498	0.19674	0.59105	0.03030
19	0.00488	0.18634	0.47843	0.02392
20	0.00494	0.17690	0.38445	0.01538
21	0.0043	0.16888	0.31229	0.02313
22	0.00464	0.16085	0.25765	0.01323
23	0.00462	0.15329	0.20940	0.01343
24	0.00458	0.14773	0.17118	0.01756
25	0.00478	0.14126	0.13843	0.00680
26	0.00472	0.13589	0.10740	0.00625
27	0.00462	0.13113	0.07927	0.01189
28	0.00455	0.12651	0.05440	0.01272
29	0.00425	0.12282	0.03347	0.02790
30	0.00378	0.11945	0.01927	0.04161
31	0.00279	0.11553	0.01039	0.05159
32	0.00182	0.11152	0.00542	0.04508
33	0.00199	0.10757	0.00292	0.00849
34	0.00191	0.10519	0.00169	1.28443E-8
35	0.00190	0.10277	9.70211E-4	1.65978E-8
36	0.00181	0.10042	6.04720E-4	4.61306E-7
37	0.00196	0.09748	3.72195E-4	9.18067E-7
38	0.00300	0.09515	2.36369E-4	1.29104E-6
39	0.00643	0.09295	1.57544E-4	2.44520E-6
40	0.01045	0.09067	1.09307E-4	3.12105E-6

Supplementary Table 6 | SA-CASSCF/RASSI-calculated electronic states for **Dy-SMM**.

Energy (cm^{-1})	Energy (K)	g_x	g_y	g_z	g_z Angle (deg.)	Wavefunction
0	0	0.00	0.00	20.00	--	99.3%|±15/2>|
350	504	0.03	0.05	17.29	3.4	98.5%|±13/2>
470	677	0.52	1.91	17.87	89.6	39.7%|±1/2> + 35.9%| ∓ 1/2> + 15.1%|±3/2>
543	782	2.94	5.24	10.56	87.9	62.1%|±3/2> + 17.7%| ∓ 1/2>
574	825	0.38	0.44	14.13	14.8	89.5%|±11/2>
611	879	0.26	4.95	6.93	18.0	71.0%|±5/2>
674	969	1.19	4.42	10.00	31.1	42.5%|±9/2> + 22.5%| ∓ 7/2> + 17.0%|±7/2>
692	996	1.41	5.73	12.70	43.8	45.8%|±7/2> + 43.5%|±9/2>

[a] Only components with a > 10% contribution are listed, with values rounded to the nearest percent.

Supplementary Table 7 | Best fitting results obtained using the generalized Debye model for the magnetic relaxations with magnetic and electric fields perpendicular to the (0-11) plane for a single crystal of **Dy-SMM** under an electric field.

T (K)	χ_S	χ_T	τ	α
20	5.1496E-23	0.82707	0.56014	0.32064
21	5.8704E-23	0.80514	0.5083	0.33006
22	7.67999E-23	0.77027	0.41756	0.3181
23	7.45261E-23	0.73526	0.33607	0.31601
24	8.52051E-23	0.72578	0.29862	0.32915
25	7.46202E-23	0.67906	0.22039	0.29769
26	9.75583E-23	0.63626	0.16828	0.28968
27	1.44677E-22	0.59776	0.12421	0.25321
28	2.26135E-22	0.58654	0.09248	0.25256
29	1.79111E-22	0.55013	0.0658	0.2156
30	2.41928E-22	0.53224	0.03795	0.2075
31	3.27774E-22	0.51369	0.02334	0.19816
32	4.88211E-22	0.49568	0.01423	0.20751
33	6.66606E-22	0.48518	0.0084	0.20863
34	9.47991E-22	0.46247	0.00466	0.18171
35	6.89560E-22	0.45237	0.00274	0.18425
36	9.41441E-22	0.43717	0.0016	0.17047
37	1.70262E-21	0.42122	9.4981E-4	0.16095
38	2.25976E-21	0.41479	5.89152E-4	0.15928
39	2.94568E-21	0.40092	3.65114E-4	0.14525
40	3.14711E-21	0.38408	2.31888E-4	0.11526
41	4.23594E-21	0.38147	1.54665E-4	0.12176
42	6.46533E-21	0.37043	1.02437E-4	0.10158
43	9.12612E-21	0.36288	7.08249E-5	0.10085
44	1.35496E-20	0.35105	5.02305E-5	0.06621
45	1.57759E-20	0.34785	3.28988E-5	0.10607
